# Supplementary material for: Are carbon emissions trading and green financial instruments synergistic? -Comprehensive quantitative research based on content analysis
Source: PLoS One. 2024 Mar 7;19(3):e0298601. doi: 10.1371/journal.pone.0298601 (PMC10919601; doi:10.1371/journal.pone.0298601)
Supplement: S1 Data — (ZIP) [file pone.0298601.s001.zip › Data/地级市Z_F工作报告.zip!/╕╩╦α/╝╬╙°╣╪╩╨/└┤╫╘╣1⁄2╓┌║┼í╛┬φ┐╦╩2╛▌═°í┐_2014.docx]

各位代表：

　　现在，我代表市人民政府向大会作工作报告，请予审议，并请政协委员和列席人员提出意见。

　　2013年工作回顾

　　过去的一年，面对严峻的经济形势，市政府在省委、省政府和市委的坚强领导下，认真贯彻党的十八大和十八届二中、三中全会精神，牢牢把握科学发展主题，加快转变经济发展方式，坚持稳中求进、好中求快的总基调，攻坚克难、奋力拼搏，较好地完成了市九届人大二次会议确定的主要目标任务，经济社会呈现平稳较快发展的良好局面。预计完成生产总值239.3亿元，增长18%（同口径比）；地区性财政收入40.3亿元，增长10.2%；市级财政收入18.3亿元，增长21%；社会消费品零售总额38亿元，增长15%；城镇居民人均可支配收入25530元，增长16%；农村居民人均纯收入12800元，增长16.5%；居民消费价格总水平涨幅控制在了3%以内。

　　一年来，我们始终坚持把项目建设作为推动转型跨越的主攻方向，发展的质量和效益有了新的提升

　　突出地企联动，强化要素保障，优化服务机制，全力推进“3341”项目工程。实施的224个项目中，百亿元以上项目2个，20亿元以上项目7个，10亿元以上项目17个。酒钢嘉酒风电基地载能特色铝合金节能技改2×45万吨电解铝项目建成投产，嘉西光伏产业园建成和在建光伏电站总装机突破100万千瓦，华强文化科技产业基地主体工程完成60%。全社会预计完成固定资产投资140亿元，增长50.1%。

　　深入实施工业强市战略，工业经济在克服困难中继续保持较快增长，实现增加值174亿元，增长20%。酒钢循环经济和结构调整、煤电铝一体化等重点项目顺利实施。广银铝业有色冶金新材料一期、索通公司预焙阳极一期、宏达公司新型干法水泥、大友公司硬质炭黑等17个重点项目全部达产达标，中威斯高精冶金新材料加工项目开工建设。科技创新能力不断加强，实施重点技术应用项目30多项，全社会研发经费占GDP比重达到2%。经济技术开发区承载能力不断提升，经济总量跃居全省省级开发区首位。

　　加快发展第三产业，促进文化旅游商贸融合发展，城市休闲消费功能不断增强。嘉峪关世界文化遗产保护与展示工程顺利推进，长城本体保护、关城修缮等重点项目已完成工程量的70%。第九届国际铁人三项赛顺利举办，大型音舞诗画情景剧《天下雄关》在嘉峪关大剧院成功首演。积极引导扶持社会资本发展文化旅游产业，相继建成了雄关冰雪文化产业园、雄关博物馆等民营文化旅游项目。旅游标准化试点工作取得阶段性成果。东方百盛购物中心投入运营，天诚国际购物中心、华城富力城市综合体等大型商贸项目启动实施。全年旅游人数达到366.8万人次，增长30.3%；实现收入23.4亿元，增长44.6%。第三产业预计实现增加值53亿元，增长15%，占GDP比重较上年提高5.3个百分点，其中文化产业增加值2.9亿元，增长36.1%。

　　坚持以工促农、以城带乡，农业产业化水平有了新提升。正大现代农业合作示范项目和标准化养殖小区建设加快推进，农村专业合作社不断壮大。“联村联户、为民富民”行动扎实开展，帮助农村居民办实事400余件，确定合作项目220项。预计完成农业增加值4.3亿元，增长9%。

　　一年来，我们始终坚持生态立市战略，人居环境进一步改善

　　坚持规划建绿、戈壁造绿、见缝插绿，完成了关城景区、工业园区、城市周边及道路两侧园林景观等绿化工程，实施了讨赖河生态治理四期项目，启动了华夏龙园建设，全年新增绿地201.7公顷，建成区绿化覆盖率达到37.5%。深入开展大气、粉尘、噪声等环境污染整治专项行动，在全省率先建成PM2.5、臭氧和一氧化碳等大气污染物自动监测系统，各项污染物排放指标控制在国家确定的目标范围内。严格抓好节能降耗，单位生产总值能耗降幅达到4.46%，单位工业增加值用水量降幅达到5%，完成了省政府下达的指标任务。

　　着力抓好基础设施建设，城市功能更趋完善。编制实施了城市风貌和色彩规划，在建筑中融入历史文化元素，地域特色不断彰显。旧城区道路、管网、绿化、亮化等基础设施改造提升力度加大。南市区基础设施配套工程和华电、东兴等企业生活基地建设加快推进。城乡一体化建设扎实开展，农村文化、体育、环卫等公共服务设施不断完善，新农村社区试点建设方案编制工作进展顺利，公共服务向农村延伸取得新的进展。

　　不断加大交通设施建设力度，区域通行能力进一步提升。嘉金公路建设加快推进，西沟矿公路和连霍高速嘉峪关收费站改扩建工程顺利竣工。兰新铁路第二双线嘉峪关段、嘉峪关南站及站前广场项目进展顺利。新开通了至重庆、广州、昆明、银川、乌鲁木齐等城市的航线，进出港航线达到14条，通航城市达到12个，旅客吞吐量突破35万人次，同比增长29.6%，跃居全省各机场第二位。

　　一年来，我们始终坚持把保障民生作为政府工作的重中之重，公共服务体系更加完善

　　坚持财政投入持续向民生领域倾斜，民生支出占财政支出的71%。高度重视社会保障工作，养老、医疗、工伤、失业、生育等社会保险保障水平稳步提升。城镇新增就业7100人，登记失业率控制在了2.7%以内。不断加大保障性安居工程建设力度，新建各类保障性住房1700套，完成棚户区改造190套。扎实推进社会救助、社会福利、慈善等工作，残疾人、重大疾病患者等特殊困难群体的生活条件得到了进一步改善。居家养老受益老人达到1.7万人，日均服务超过1300人次。加大公共交通投入，新增公交车14辆、校车6辆，城市公交线路累计达到11条，群众出行更加方便。各级各类教育快速发展，义务教育巩固率达到99%，高中阶段毛入学率达到94%，高考录取率达到90.4%。医药卫生服务体系进一步健全，重点传染病防控成效明显，居民家庭健康档案基本建立，卫生信息化建设水平大幅提升。新农合参合率达到98.8%。社区文化休闲设施进一步完善，群众性文化体育活动深入开展，成功创建全国全民健身示范城市。全国文明城市创建工作取得阶段性成果。

　　立足全市人民所想所盼，全面完成了12件惠民实事。（1）实现了60岁以上老人免费乘坐公交车，惠及3.1万老人。（2）对阳光、昌盛、朝晖3个居民小区进行了综合改造，改善了居民生活环境。（3）市酒钢三中新校区、市四中综合教学楼主体工程完工；市一中、六中综合教学楼和5所学校操场改造、7所农村中小学食堂建设全部竣工投入使用。（4）市中医院改造工程和新城镇、文殊镇卫生院建设项目顺利完成；妇幼保健院二期工程主体完工；市第一人民医院迁建及儿童病区建设完成各项前期工作；为全市所有参合农村居民进行了免费体检。（5）开展了居家养老日间护理上门服务，免费接送就医和上门医疗服务达到1100人次。（6）建成了公共自行车租赁系统，配备自行车150辆，设置服务点8个，实现了通借通还。（7）新建了3台智能图书借阅机和10个电子阅报栏，方便了群众学习阅读。（8）完成了农村中心敬老院、儿童福利院、社会福利院三院联建及残疾人康复托养中心建设。（9）新建、改造了13个城乡标准化社区服务中心，全市标准化社区服务中心达到了20个。（10）建成了10家农超对接平价店和25户市级食品安全示范店，为周边居民生活提供了便利。（11）新建了公共卫生间10座并全部免费开放，在方便群众的同时提升了旅游城市形象。（12）建成了市政务服务中心和公共资源交易中心，实现了集便民服务、行政审批、公共资源交易和电子监察四位一体的一厅式办公和一站式服务。

　　全面深化社会治理，平安嘉峪关建设有序推进。全年刑事案件发案率同比下降23.5%。在全省率先实施了律师进社区服务活动，面向基层和群众的法律服务不断加强。集中排查和化解矛盾纠纷，群众反映的合理诉求得到有效解决。整治环境卫生，加强和改进物业管理，开展宠物饲养专项治理，维护了公共环境卫生和秩序，城市管理效能进一步提升。高度重视安全生产，集中开展安全生产大检查，修订完善了各领域应急预案，定期开展防震减灾应急演练，安全形势持续稳定。不断加强对食品安全、药品安全、物价等市场监管，有力地维护了市场秩序，社会保持和谐稳定。

　　一年来，我们始终坚持推进改革开放，经济社会发展活力不断增强

　　紧紧围绕经济社会发展中的体制性问题，不断深化改革，在一些重点领域和关键环节取得新的突破。搭建了文化旅游、水文化、经济开发区、城市基础设施投资开发、农业发展等五大平台，通过盘活存量资产融资近30亿元。深入开展政银企融资对接活动，不断扩大信贷投放总量，全市银行业金融机构各项贷款余额达到353.2亿元，增长18.6%。完成了工商、质监、食品药品监督管理体制改革，监管和服务效能进一步提升。全面落实促进非公经济发展的各项政策措施，非公经济完成增加值43亿元，增长20%。文化、水利、财税、城乡户籍制度等改革加快推进，发展活力不断增强。

　　立足区位和发展优势扩大开放，内引外联取得积极成效。支持酒钢公司启动与新疆广汇能源合作建设煤炭分质利用项目。与张掖联合开发七一冰川、文殊寺等旅游资源取得重要进展。航空口岸开放和保税物流中心申报工作正式启动。招商引资取得重大突破，成功引进深圳华强、正大集团、中国文化传媒、浙江正泰等知名企业在我市投资兴业。招商引资省外到位资金103亿元，同比增长119.3%。

　　一年来，我们始终坚持把提升行政效能作为政府自身建设的关键环节，作风建设取得明显成效

　　坚持依法行政，自觉接受人大法律监督、政协民主监督和社会公众监督。全年共办理市人大代表意见建议75件、政协提案和建议101件。坚持科学民主决策，认真执行重大事项集体决策、专家咨询、社会公示与听证制度，政务公开和政府信息公开深入推进。强化行政执法监督，行政权力运行更加规范。认真办好“12345”民生服务热线，坚持“市长接待日”制度，解决了一批涉及群众利益的热点难点问题。

　　不断创新服务方式，深化效能风暴行动，取消行政许可、审批项目61项，审核并向社会公示收费项目434个。将所有公共资源交易业务和行政审批事项纳入政务服务中心统一办理，规范了交易行为，提高了办事效率，审批时限平均压缩45%。对政府投资项目全部实行“代建制”管理，提高了项目建设的专业化水平和投资效益。

　　认真贯彻落实中央“八项规定”、省委“双十条规定”和市委“七项规定”，开展了办公用房、公务用车清理和会员卡清退工作，坚决遏制奢侈浪费和公款送节礼等不正之风，文风会风和政风行风进一步好转，“三公经费”开支较上年压缩39%，全部用于民生支出。严格落实党风廉政建设责任制，扎实推进惩治和预防腐败体系建设，深入开展廉政风险防控，强化行政监察和审计监督，从源头上遏制了违纪、违法案件的发生，维护了为民、务实、清廉的政府形象。

　　此外，粮食、供销、电力、邮政、通信、人防、统计、档案、地方志、气象、地震、民族宗教、外事侨务、科普、国防教育、普法、人口与计划生育、妇女儿童等工作也都取得新的进展。

　　各位代表，过去的一年极不平凡，取得的成绩来之不易。这是省委、省政府亲切关怀，市委坚强领导，市人大、市政协监督支持，嘉峪关军分区、酒钢集团公司、中核四○四有限公司及驻嘉各单位共同努力，全市广大干部群众共同奋斗的结果。在此，我代表市人民政府，向全市各族人民，向人大代表、政协委员、各民主党派、人民团体、社会各界人士和离退休老同志，向驻嘉部队、武警消防官兵、驻嘉各单位表示崇高的敬意和衷心的感谢！

　　在看到成绩的同时，我们也清醒地认识到，全市经济社会发展中还存在一些突出的矛盾和问题，主要表现在：三次产业结构不协调，第三产业比重偏低，经济结构调整任务依然艰巨；生产要素和资源环境约束趋紧，节能减排和环境保护压力进一步加大；部分民生问题的解决与群众的要求还有差距，社会服务和治理体制还需要进一步创新；少数政府部门工作人员作风不实、效率不高、开拓创新意识不强等问题还不同程度存在。这些问题，既是制约我市当前经济社会发展的难点，也是我们做好下一步工作的重点，我们将高度重视并采取有效措施，认真加以解决。

　　2014年主要工作

　　2014年是贯彻党的十八届三中全会精神、全面深化改革的开局之年，也是落实市第十次党代会精神、实施“十二五”规划的攻坚之年。市政府工作的总体要求是：深入贯彻党的十八大和十八届二中、三中全会，省委十二届六次全委会及全省经济工作和城镇化工作会议精神，按照市委十届四次全委扩大会议的决策部署，紧紧围绕率先建成全面小康社会的奋斗目标，坚持稳中求进的工作总基调，把改革创新贯穿于经济社会发展各个领域，更加注重激发市场活力，更加注重转方式调结构，更加注重加强基本公共服务体系建设，更加注重改善民生，不断提高经济发展质量和效益，着力促进经济持续健康发展、社会和谐稳定。

　　全市经济社会发展主要预期目标是：地区生产总值增长12.5%，市级财政收入增长13.5%，城镇居民人均可支配收入增长13%，农村居民人均纯收入增长13.5%，全社会固定资产投资增长30%，规模以上工业增加值增长14.5%，社会消费品零售总额增长14%，居民消费价格总水平涨幅控制在3.5%以内，单位生产总值能耗和主要污染物排放完成省上下达的控制指标。

　　上述目标是在综合分析经济形势和改革发展任务，统筹考虑稳增长、抓改革、促调整、惠民生、防风险等各方面因素的基础上提出的。核心目的就是要把经济增长保持在合理的区间内，更加注重提升经济发展的质量和效益，更好地推进结构调整、保障和改善民生、促进社会和谐稳定，为实现全市经济社会持续健康发展奠定坚实基础。经济增速较上年虽有所下调，但我们加快发展的决心不会改变，转型跨越的措施不会松劲，争先进位的奋斗不会停歇。我们将切实转变发展观念，紧紧抢抓发展机遇，牢牢把握工作主动权，努力做到稳中有为、稳中提质、稳中增效，切实增强发展的协调性、均衡性和可持续性。

　　围绕顺利实现上述奋斗目标，着重抓好以下几个方面的工作：

　　一、突出产业结构调整，促进经济转型升级

　　坚持把结构调整作为推动转型跨越的攻坚重点，深入推进“3341”项目工程，着力优化投资结构，提高投资效益。抓住用好“丝绸之路经济带”、老工业基地调整改造、华夏文明传承创新区和国家生态安全屏障综合试验区等战略平台，将我市的功能定位、产业发展规划和基础设施建设融入全省战略布局，谋划、实施、建成一批重大项目，为全市经济社会发展提供有力支撑。进一步强化服务保障，提高审批审查效率，扎实推进项目建设。深入开展政银企对接活动，持续加大信贷投入力度，继续扩大直接融资，鼓励社会资本投资项目建设。加快推进招商引资项目落地实施，努力提高项目履约率和资金到位率。

　　坚持用高新技术改造提升传统优势产业和培育战略性新兴产业双轮驱动，推进工业结构调整。加快我市省级清洁能源及冶金有色新材料循环经济基地建设，全力支持酒钢公司循环经济和结构调整、嘉北新区煤电铝一体化、煤炭分质利用等重点项目建设，推进广银有色冶金新材料加工二期、索通预焙阳极二期、中威斯高精冶金新材料加工、嘉西光伏产业园光伏电站、110千伏汇集升压站及送出工程等重点项目，着力构建“冶金-循环经济-装备制造”和“光伏发电-电解铝-铝制品加工”两条千亿级产业链。大力实施创新驱动战略，推动各类创新要素向企业项目聚集。继续完善经济技术开发区道路、管网、绿化等基础设施，增强承接产业转移能力。

　　全力推动文化、旅游等第三产业发展提速、比重提高、水平提升。继续抓好世界文化遗产保护与展示工程、华强文化科技产业基地、丝绸之路非物质文化遗产古镇、观礼古镇、七一冰川、戈壁大峡谷地质公园、草湖国家湿地公园、华夏龙园、“长城边关第一镇·丝路印象”等文化旅游重点项目建设，巩固提升全国旅游标准化试点成果，加快嘉峪关大景区规划建设，打造特色文化产业集群。办好铁人三项世界杯赛，加大城市对外宣传力度，促进文化旅游深度融合。全面落实国家深化流通体制改革、支持现代服务业发展的政策措施，推进天诚国际购物中心、华城富力城市综合体、恒基美居文化广场和五一商城、雄关宾馆改造等重点商贸项目建设，努力打造新华路核心商贸聚集区。加快编制城市商业网点规划，推动社区便民商业网点建设改造，进一步完善城乡市场体系，满足居民消费需求。

　　二、坚持走新型城镇化道路，努力建设生态宜居美丽雄关

　　坚持以人为本，立足市情，推进以人为核心的城镇化。进一步完善城乡总体规划，科学布局生态、生产、生活功能分区，优化城市发展空间，合理限定城市规模边界，切实提高城镇建设用地集约化程度。坚持一张蓝图绘到底，维护规划的严肃性和权威性。保护和弘扬传统优秀文化，延续城市历史文脉。科学制定新的户籍管理办法，有序引导增量人口落户，促进稳定就业和生活的常住人口实现市民化，让所有在嘉峪关工作生活的建设者享受城市发展成果。

　　强化城市建设管理，提升居民生活质量。加快推进南市区基础设施配套工程，稳步实施建成区道路、停车场、给排水、供热、供气等基础设施建设，逐步改善老城区居民小区环境，协调各方力量加快推动棚户区改造，鼓励社会资本参与城市公共设施投资运营。完成嘉金公路建设和黑鹰山战备公路改建工程，积极推进312国道绕城道路和省道215线提级改造项目，建成兰新铁路第二双线嘉峪关南火车站及站前广场，积极推动嘉峪关机场改扩建，加快建设区域大交通格局。开展物业管理年活动，不断理顺管理体制，规范行业行为。推进道路交通秩序和市场、市容环境综合整治，改善群众生活环境。切实加强市民文明素质教育和思想道德建设，扎实开展群众性精神文明创建活动，积极推进无烟机关建设，努力争创全国文明城市。深入推进“智慧雄关”和全国信息消费试点城市建设，充分运用“数字嘉峪关地理空间框架”成果，提升城市管理精细化、信息化水平。

　　坚持规划、政策、产业、环境并重，加快城乡一体化建设进程。修订农村新型社区规划，按照产居分开、人畜分离的原则，科学布局住宅、生产、养殖区域。启动安远沟城镇化试点工程。落实各项强农惠农政策，深入推进城乡社会保障政策统筹衔接，积极稳妥推进农村土地承包经营权流转，探索建立宅基地退出和补偿机制。把城镇化与育产业、促就业紧密结合起来，引导和鼓励农村居民从事非农产业，发展具有田园特色的旅游观光和休闲度假产业。加快正大现代农业合作示范项目建设进度。推进产业化种植基地和标准化特色养殖小区建设，发展壮大农村专业合作组织，千方百计促进农村居民持续增收。健全运行保障机制，确保农村道路、环卫、照明等基础设施正常运转。

　　坚持生态优先，以更大的决心和力度推进生态宜居城市建设。修订完善城乡生态建设总体规划，推进城乡生态一体化进程。重点实施新华路、和诚路、迎宾路、雍和街区等道路、小区的绿化改造提升工程，抓好城乡生态防护林建设，不断提高园林化、景观化水平。支持酒钢集团公司国家级循环经济试点工作，狠抓企业节能降耗，坚决淘汰落后产能，全面促进资源节约和综合利用。强化环境污染综合治理，抓好重点企业污染物减排工作，严格控制污染物排放总量，努力建设美丽雄关。

　　三、着力保障和改善民生，推动社会事业均衡发展

　　坚持民生优先，进一步优化财政支出结构，压缩公共服务支出，把更多的财力用于保障和改善民生。深入实施全民创业工程，年内新增就业7500人以上，城镇登记失业率控制在2.7%以内。推进各类用人单位参加工伤保险和生育保险，按照省上统一部署全面推开城乡居民大病保险，进一步巩固提升城乡居民养老保险和医疗保险保障水平，参保率巩固在95%以上。进一步完善住房保障和供应体系，加快城乡保障性住房建设，努力改善困难群众住房条件。全面落实各项社会救助制度，高度重视困难职工、老年人、残疾人、重大疾病患者等特殊困难群体的生活，支持慈善公益事业加快发展。

　　大力实施教育、医疗、文化等惠民工程。实施市第三、第四幼儿园加固改造及扩建工程，建成市酒钢三中新校区、市四中综合教学楼，进一步改善办学条件，推进义务教育均衡发展。深化医药卫生体制改革，优化调整全市各类综合医院布局，建成市计划生育服务中心，加快培养社区卫生服务中心全科医生队伍，构建综合医院、基层医疗机构和流动上门就诊相结合的医疗服务体系。提高重点传染病防控和突发公共卫生事件应急处置能力，做好国家卫生城市第二次复审工作。加快推进社区文化体育活动场地和乡村舞台建设，让文艺精品进社区、近市民。

　　全力推进平安中国示范区建设。继续加强网格化管理和社会化服务，创新立体化社会治安防控体系，严密防范和依法打击各类违法犯罪活动。完善“12345”民生服务热线、民情流水线和社会治理服务综合信息平台功能，加强以“三大中心”为依托的新型治理服务体系建设。创新社会矛盾预防化解机制，健全重大决策社会稳定风险评估制度，深入开展律师进社区活动，及时化解各类矛盾纠纷。管好用好价格调控基金，加强价格监管和“菜篮子”工程建设，确保市场供应与物价水平持续稳定。加大设施设备投入，健全食品安全源头治理和长效监管机制，全力保障食品药品安全。进一步强化安全生产责任体系建设，扎实开展应急救援演练，认真做好安全预防控制，坚决遏制重特大安全事故发生，确保安全生产主要指标不突破控制红线。

　　2014年，重点办好以下惠民实事：（1）实行全市所有残疾人免费乘坐公交车，为重度残疾人发放护理补贴，对200户重度残疾人家庭进行无障碍设施改造。（2）完善居家养老服务平台建设，实现居家老人上门医疗服务城乡全覆盖。（3）完成市二中、新城中学、新城镇中心小学教学楼加固改造，启动建设明珠学校初中部。（4）强化社区服务功能，新建改造6个城乡标准化社区，配套建设3个农超对接超市，建成9个体育健身场地。（5）改善旧城区人居环境，对兰新、昌明居民小区进行环境综合改造。（6）实施广电网络双向改造一期工程，完成有线电视用户改造5万户。（7）在新老城区建设公共卫生间10座，并免费开放。

　　四、全面推进体制机制创新，不断开创改革开放新局面

　　深入贯彻落实党的十八届三中全会精神，按照中央、省委、市委关于全面深化改革的安排部署，抓好各项改革措施的细化落实，为全市经济社会发展提供强大动力和体制保障。深化经济体制改革，着力推进国有资产和国有企业管理体制改革，大力发展混合所有制经济，加强国有资产管理，保障职工权益。全面落实国家宏观调控政策，深化投资体制改革，确立企业投资主体地位。坚决破除非公经济行业准入的各种障碍，全力支持非公经济加快发展。深入推进财税、城乡一体化、社会事业、社会治理等方面体制机制改革，最大限度激发发展活力。

　　加快转变政府职能，强化公共服务、市场监管、社会治理、环境保护等职责。深化行政审批制度改革，进一步划清政府与市场的边界，充分发挥市场在资源配置中的决定性作用，突出做好“接、放、管”，接好中央、省上下放的审批事项，放出该放的权力，管好该管的事情。对保留的行政审批事项进一步进行规范，统一纳入政务服务中心集中办理，简化审批环节，提高审批效率。稳步推进政府机构改革，严格控制机构编制，确保财政供养人员只减不增。有序推进事业单位分类改革，加大政府购买公共服务力度。完成卫生和计生机构整合工作。

　　牢牢抓住共建“丝绸之路经济带”的历史性机遇，进一步拓展同周边地区和中亚、西亚国家的经济合作，加快向西开放步伐。积极推动设立嘉酒哈循环经济及能源综合利用合作示范区，加快推进航空口岸开放和嘉峪关保税物流中心建设，规划建设嘉峪关国际港务区。积极承接东部地区产业转移，推进大项目招商、产业链招商，着力引进一批符合国家产业政策、科技含量高、投资强度大的大项目、好项目。

　　五、扎实推进作风建设，努力建设为民务实清廉政府

　　坚持依法行政，建设法治政府。自觉接受人大法律监督和政协民主监督，虚心接受舆论监督和群众监督，认真办理人大代表建议和政协提案。深入推进政务公开和政府信息公开，健全重大决策社会公示和听证制度，主动听取各方面意见，不断改进政府工作。严格按照法定权限和程序行使职权、履行职责，切实规范执法行为，加强执法监督，让权力在阳光下运行。

　　坚持改进作风，建设服务政府。牢固树立为民服务理念，继续加大软环境整治力度，进一步提高办事效率，增强为企业、为基层、为群众服务的能力和水平，凝聚各方面力量，努力创造风清气正、公正开放的发展环境。大力弘扬开拓创新、务实高效的工作作风，以踏石留印、抓铁有痕的决心，狠抓各项工作落实，不断提高政府的执行力和公信力。

　　坚持清正廉明，建设廉洁政府。严格执行中央、省委、市委作风建设各项规定，切实改进会风文风，树立良好政风。继续推进办公用房和公务用车清理，在全市所有预算单位开展预决算及“三公经费”公开工作。严格执行党风廉政建设责任制，加强廉政风险防控，进一步健全政府重点投资项目监管和全程跟踪审计制度，切实做到干部清正、政府清廉、政治清明。

　　坚持群众路线，建设民生政府。扎实开展党的群众路线教育实践活动，坚决纠正“四风”问题，从人民群众最期盼的事情做起，从人民群众最不满意的地方改起。牢固树立群众观点，时刻牢记群众想的就是我们要干的、群众的难点和热点就是我们工作的重点，尽心竭力办实事、解难题，用我们的辛苦指数换取老百姓的幸福指数，努力建设对人民负责、为人民造福、让人民满意的政府。

　　各位代表，改革创新任重道远，新的征程催人奋进。人民群众对美好生活的新期待，始终是我们坚持不懈的奋斗目标。让我们在省委、省政府和市委的坚强领导下，锐意进取、埋头苦干，为率先实现经济社会转型跨越发展，建设幸福美好嘉峪关而努力奋斗！
